# Prof. Hamilton’s Fracture Tables

**Published:** 1849-05

**Authors:** 


### PROF. HAMILTON'S FRACTURE TABLES.

Prof. Hâmilton, of Buffalo, has collected and thrown into tabular form the history of 136 cases of fractures, for the purpose of determining the average results of treatment in such casualties. He does not believe that the results are equal to the expectations of the friends of surgical science. His tables will be examined with great interest by the surgeon, since it is only from such general statistics that safe opinions can be derived. The following is his summary of the various fractures:

*Ossa Nasi*, three cases; of which none are perfect; there being more or less deformity in each case.

*Vomer*, one case, with lateral displacement.

*Inferior Maxilla*, whole number five; all in adults; all compound comminuted; all have united. Three are perfect and two imperfect.

*Clavicle*, total eighteen. Perfect six; imperfect twelve. Two of the six perfect cases are children under eight years. Fifteen occurred near the junction of the outer third with the inner two-thirds; three near the middle. Four were comminuted; fourteen simple. I believe not more than three or four were transverse. All have united. Eight shortened from a quarter of an inch to an inch.

*Acromion Process*, one case; united by ligament, but use of arm perfect.

*Humerus*, fourteen. Twelve simple; two compound comminuted; thirteen united, one not united; three shortened from half to three-quarters of an inch. Five perfect; nine imperfect.

*Radius*, eight. All simple. Five perfect; three imperfect.

*Ulna*, nine. Seven simple; two compound. Eight perfect; one imperfect.

*Radius and Ulna*, thirteen. Eleven simple, one compound, and one comminuted. Two not united. Seven perfect; six imperfect.

*Ribs*, four. All simple. Two perfect; two imperfect.

*Posterior Superior Spinous Process of Ileum*, one case, and this left the fragment projecting.

*Femur*, twenty-eight. Seven through the neck; whether within or without the capsule I have not determined in but one instance. The signs are generally too obscure to make a positive diagnosis. Twenty-six simple; two comminuted. Of twenty broken through the shaft, fourteen are shortened from half an inch to two inches. Only two fractures of the shaft of the femur, out of fifteen cases occurring in adults, are perfect. Probably all the fractures through the neck are shortened, but they were not all measured since recovery.

*Patella*, three. All united by ligament.

*Fibula*, four. Two perfect; two imperfect.

*Tibia*, two. One perfect; one imperfect.

*Tibia and Fibula*, twenty-two. Seven simple; fifteen compound, or comminuted, or compound and comminuted. Nineteen united; three not united. Twelve shortened from quarter of an inch to an inch and a half. Five perfect; seventeen imperfect.

